# ‘An experiment pervyd for a thynge y lost’: ‘Non-medical’ Charms and *experimenta* in Medieval Medical Manuscripts

**DOI:** 10.1093/shm/hkae053

**Published:** 2025-04-15

**Authors:** Heather A Taylor

**Affiliations:** University of Kent, Rutherford College, Canterbury CT2 7NX, UK

**Keywords:** medieval, manuscripts, charms, practitioners, magic

## Abstract

This essay conducts a close examination of manuscripts of English provenance from the late Middle Ages which, while predominantly medical in nature, also contain non-medical charms and *experimenta*. It considers how these manuscripts might provide evidence for a particular type of medical practice, one which was founded in text-based learning, but which also sought to address the non-medical concerns and anxieties of medieval patients through the application of charms and *experimenta* not exclusively related to healing. This enables a more detailed picture to be drawn of medical practice in the Middle Ages but, more specifically, of medical practice within a particular stratum of society, whereby patients or clients may have looked to engage the services of a practitioner whose literacy and text-based knowledge afforded him status, but who also addressed issues that were perhaps more commonly treated by humbler diviners and healers.

*Ut carcer aliquem non possit detinere. Vade ad herbam que dicitur celedonia et collige eam in vigilia Sancti Petri dicens Pater Noster et ave in honore Dei et Sancti Petri ad Vincula et portet super te et carcer te non retinebit. Experimentum est probatum*

So that a prison cannot hold someone. Go to the plant that is called celandine and collect it on the eve of the feast of Saint Peter saying the *Pater Noster* and *Ave* in the grace of God and St Peter ad Vincula and carry it on you and no prison will hold you. This experiment is proved.^1^

This experiment to evade captivity features in a tract of medical recipes that forms part of the *Liber medicinarum*, ascribed to the well-known fourteenth-century surgeon John Arderne. It precedes recipes for a pill to aid sleep, and to relieve arm pain. No modifications to the *mise-en-page*, scribal variation or different methods of rubrication or punctuation indicate that this experiment is considered different from the recipes that surround it, and yet its utility seems out of place in a manuscript of otherwise exclusively medical content.^2^

Charms and *experimenta* for healing were, at best, ignored, and at worst, derided by nineteenth and twentieth-century scholars of medieval medicine.^3^ However, more recently, much-needed work has been done to validate their place in the wider medical corpora of texts. Through an examination of manuscripts that bear witness to the medical practice of established physicians and surgeons, including John Arderne, Lea Olsan has demonstrated that ‘magical’ healing was not just the preserve of ‘folk’ or ‘popular’ medical practitioners, but was integrated into the services of—even university trained—physicians who practised at court.^4^ The study of how charms complement or enhance the offerings of medical practitioners has helped to develop a more comprehensive understanding of medical practice in the Middle Ages, as well as a more nuanced consideration of magic and its uses in the period. But while healing charms have now been fairly widely studied, the non-medical charms and *experimenta*—like the one to free a prisoner cited above—which frequently appear alongside recipes or embedded into treatises have been relatively ignored. Further examination of these non-medical elements in medical manuscripts can undoubtedly further both our understanding of services rendered by the medieval medical practitioner, as well as the uses, status and monetisation of magic within the period.

The present study will perform a close examination of English manuscripts from the late Middle Ages (c. 1100–1500) which, while predominantly medical in nature, also contain non-medical charms and *experimenta*. In doing so, it will consider how these manuscripts might provide evidence for a particular type of medical practice, one which was founded in text-based learning, but which also sought to address the non-medical concerns and anxieties of medieval patients through the application of charms and *experimenta* not exclusively related to healing. This will draw a more detailed picture of medical practice in the Middle Ages but, more specifically, of medical practice within a particular stratum of society, whereby patients or clients may have looked to engage the services of a practitioner whose literacy and text-based knowledge afforded him status, but who also addressed issues that were perhaps more commonly treated by humbler diviners and healers.

## Terminology

It is not possible to go any further without devoting some space to a consideration of the terminology in use here. While I have already succumbed to the temptation to use the word ‘magic’ above, it has long been acknowledged as a problematic term. In particular, it has been used by modern scholars in ways that are anachronistic, but that serve to encompass a broad range of practices, including demon-summoning and necromancy, charms and natural magic.^5^ Recently, Richard Kieckhefer has noted that ‘magic’ can be considered an ‘aggregating term’. Aggregating terms are:

[D]ifficult to define, because they encompass diverse elements that may or may not be combined with each other. The different elements may not share any common defining feature that brings them under the umbrella of the aggregating term; they are not linked by a shared essence. They may not even have shifting combinations of shared features; they are not necessarily bound by family resemblance.^6^

In acknowledging that ‘magic’ is an aggregating term, Kieckhefer recognises that it is one of convenience, but to which we devote too much time to refining; Kieckhefer exhorts scholars to instead pay more attention to what he labels ‘constitutive terms’, ones which connote specific forms of reference.^7^ It is in this regard that I mainly confine my terminology to the description of two categories: ‘charms’ and *experimenta*, or ‘experiments’. Charms, to use Lea Olsan’s definition, are ‘spoken, chanted and written formulas, derived ultimately from a traditional oral genre and circulated both by word of mouth and through manuscript and amuletic texts’, and are designed to bring about a certain effect.^8^ ‘*Experimenta’* is a little broader. Within the context of medieval medicine, *experimenta* referred to treatments or remedies which were understood to work by experience, rather than accounted for through theory.^9^ This originated in the natural philosophy of antiquity, when authors such as Pliny the Elder and Galen recorded the occult or hidden virtues of animals and certain natural substances, which they noted could not be identified, but which were known to bring about certain effects. These effects were accounted for only through the experience of an observer, who could then attest to their efficacy.^10^ In this sense, charms fall into the category of *experimenta*, in that they had been observed to work, but their efficacy could not be explained by Galenic principles, nor by any other identifiable natural property.^11^ However, I use the term to describe rituals that did not simply, or always, rely on the power of words—whether verbal or textual—and that also incorporated objects, such as animal parts, herbs or stones, believed to possess occult properties. While many such rituals combined verbal components with other items—much like the experiment to free a prisoner in John Arderne’s text, which draws on the power of the *Pater Noster* as well as the properties of the plant celandine—other experiments utilise only the occult properties of objects, without the addition of efficacious words.^12^ I therefore deploy the term ‘charm’ to signify any method or ritual which features a standalone verbal element, whether this is an incantation or inscription, while ‘experiment’ indicates a method that incorporates this verbal element into a wider process involving objects with occult properties, or which relies only on such objects and ingredients alone. Occasionally, for convenience and to avoid frequent and clunky caveats, I will use the term ‘magic’ or ‘magical’ to refer to these charms and experiments as one entity, in distinction from the medical content that surrounds them in their manuscript contexts.

## Charms and Experiments in Medical Manuscripts

Recent scholarship has aided our understanding of the evolution, transmission, narrative backgrounds and key motifs for many medical charms. T. M. Smallwood has traced the oral and textual transmission of charms, both within England and across borders, while Lea Olsan has investigated the semantic motifs, that is, the ‘key meaning content within the incantations, or operative words, of a charm’.^13^ The justification for their status as an acceptable and licit part of medical practice has been cemented by the work of Olsan and Peter Murray Jones, and by the work of other scholars such as Catherine Rider whose exploration of medieval pastoral manuals exposes a tolerant acceptance of certain verbal cures in medical practice.^14^ Outside of medicine, Eamon Duffy has demonstrated that charms are not out of place beside more orthodox religious material in medieval prayer books and Books of Hours, and Richard Kieckhefer has outlined practices belonging to the ‘common tradition’, that is, magic which ‘was distributed widely and that was not regularly limited to any specific group’.^15^

The most comprehensive study of the conflation of medical practice with non-medical, magical elements, is Owen Davies’ work on ‘cunning folk’, which traces these figures from the early modern period through to the twentieth century.^16^ Davies builds on the work of Keith Thomas and Alan Macfarlane in describing this particular genre of practitioner, and asserts that cunning folk are defined by the services they offer, such as healing the sick and bewitched, fortune-telling, identifying thieves, recovering lost items and performing love magic.^17^ Davies’ study is useful here, in that he identifies a type of practitioner who met the multi-faceted needs of their clients through a combination of medical and magical knowledge. However, in building his profile of cunning folk, Davies often conflates evidence provided by sources from the entire breadth of his study’s chronology. This leads to overestimation of certain qualities such as literacy rates, which would have greatly differed in the twentieth century as compared to the sixteenth.

A recent study by Tabitha Stanmore builds on Davies’ work on cunning folk.^18^ Stanmore opts for the term ‘service magician’ over cunning folk, in part because the term cunning folk often brings to mind someone who practices unlearned rituals, or who might be considered a lower status, parochial figure.^19^ Stanmore’s study is complementary here; she uses documentary records such as court records and other literary sources to provide evidence of service magic in the late medieval and early modern period, and makes a number of findings relevant to the present study, including that the status of the client influenced both the status of the practitioner they consulted, as well as the type of services they commissioned.^20^ Stanmore does not include manuscript evidence in her corpus of sources, but when her findings are considered alongside the manuscript evidence presented below, we can see a number of interesting parallels that help to further flesh out a model for a specific type of practitioner during this period.

## Medical Practice in Medieval England

It is unnecessary to provide an in-depth consideration of medical practice in the Middle Ages here. However, it is helpful to summarise some key points that will prove useful when considering how a particular genre of medical practitioner may have found a niche in the market during the period. Medical practice in medieval England was diverse and difficult to define. Until the start of the thirteenth century, monastic houses were the main centre of medical learning; thereafter the accumulation and transmission of medical knowledge predominantly took place outside of the cloister.^21^ On the continent, the medical curriculum flourished at certain universities, particularly in Italy and France, and the fourteenth century saw the establishment of several medical colleges of physicians, something which did not occur in England until 1518.^22^ In England, while medicine was covered on the curriculum for those obtaining an arts degree, it did not become a subject in its own right until the fourteenth century, and even after this date the number of university-trained doctors remained fairly low.^23^

Outside of university-trained physicians, there were a plethora of other practitioners. Amongst them were physicians who had attended university without completing a medical degree, but they were in competition for clients with self-taught practitioners or ‘leeches’, barber-surgeons and apothecaries.^24^ While the university towns of Oxford and Cambridge were the centres of text-based medical learning, as the Middle Ages progressed, medical texts became increasingly available in the vernacular, meaning that those who were not university trained had access to the same body of knowledge attained through an official qualification.^25^ Whether a practitioner was university trained, self-taught through the acquisition of medical tracts and treatises, or had built their practice through skills handed down generation to generation and disseminated orally, the number of self-professed physicians who declared medical practice as their main occupation was still very low.^26^ Thus it appears that medical services were offered by many who combined such practices with other occupations. One of the very few witnesses we have to the medical practice of a known practitioner is the personal manuscript of John Crophill which contains details of the patients he treated between 1456 and 1485.^27^ As well as offering medical services, Crophill was a bailiff for the priory of Wix in Essex and an ale-taster.^28^ His medical practice provided another source of income alongside his other duties.

Further to John Crophill’s manuscript, there are only a small number of other sources which provide an insight into the actual services rendered by a medical practitioner. Highly valued for its record of patients treated is the manuscript of the fifteenth-century physician Thomas Fayreford.^29^ This list of patients tells us much about Fayreford’s medical practice: that he travelled some distance while carrying out his practice, that he treated patients from a wide range of social backgrounds, that he treated both men and women, and that he provided cures for ailments of varying complexity, from thorns, burns and fractures, to gynaecological problems, most commonly ‘suffocation of the womb’.^30^ Another manuscript that records the services of a known practitioner is that of Richard Trewythian.^31^ Like Crophill, Trewythian did not practice medicine to the exclusion of other services. His manuscript reveals that he was also a moneylender, book-dealer and astrologer. Like Fayreford, Trewythian’s notebook reveals that he treated patients from diverse backgrounds, while records of his astrological consultations reveal much about common anxieties of the period, a point to which I will return later.^32^

Further to the above manuscripts, which can be associated with a particular owner and practitioner, there are other anonymous sources that provide some insight into the realities of medical practice in the Middle Ages. Records of prescriptions made up by apothecaries in British Library, Harley MS 1628 reveal their working relationship with physicians, as well as some of the treatments administered to some very high-profile patients including Edward IV and Richard III.^33^ The inclusion of a banns advertising the services of an itinerant leech on fol. 106v of British Library, Harley MS 2390 provides a rare insight into the marketing of medical services in the fifteenth century, and sheds some light on the treatments actually utilised. In her study of this manuscript, Linda Ehrsam Voigts demonstrates that each treatment item advertised in the banns proclamation can be linked to the items of practical knowledge recorded in the rest of the codex, suggesting that the body of knowledge found in a manuscript might be a reliable indicator of the actual services administered by the practitioner, rather than functioning simply as a repository of information.^34^

We can draw several broad conclusions from these sources. Medical practice was often only one facet of a practitioner’s profession; regardless of the status of the practitioner, they treated a broad spectrum of social statuses, and that where there is a record of treatments used as well as a body of practical knowledge, the two can be sufficiently linked so as to understand the body of knowledge as a reliable indicator of the services actually administered by the practitioner. These conclusions are all useful when examining the evidence supplied by the manuscripts discussed below.

## Non-medical Charms and Experiments in Medical Manuscripts

In order to give a sense of the prevalence of non-medical charms and experiments in medical manuscripts, I will first provide a broad overview of the data by means of a quantitative breakdown, before using three case studies to support a more in-depth analysis. For the purposes of this study, I have identified a total of 48 manuscripts which, though containing almost exclusively medical material, also feature non-medical charms and experiments.^35^ All of English provenance, these manuscripts span a broad chronology, with the earliest originating in the late-eleventh century, and the latest from the early-sixteenth century. I have assessed each manuscript on a case-by-case basis, in order to ascertain whether it may have been commissioned or copied for professional or personal use. This is by no means clear for the majority of manuscripts. Medical recipes and charms were often copied into personal commonplace books or household books, and may only have been intended for use by the owner and his or her direct family.^36^ I have eliminated, from what was originally a larger corpus, any manuscripts that are identifiable as personal or household books, as well as other typical venues for charms such as Books of Hours. While we cannot say for sure if the remaining manuscripts were used—either by a physician or a less formally trained medical practitioner—their strong focus on both theoretical and practical medicine indicates that they were representative of much of the textual basis for medieval medical practice.

Within the manuscripts surveyed, I have identified at least 250 non-medical charms and experiments.^37^  Table 1 outlines the purpose of these texts and the frequency with which they occur. The categories are necessarily broad. Practices to prevent theft or to identify a thief are collapsed into one. ‘Love’ encompasses any practice to provoke or prevent both love and lust, as well as to uncover adultery, and to cause either marital harmony or discord. This creates some crossover with the category to uncover secrets, in which a number of the experiments specify that they will reveal a woman’s secrets, and likely refer to adultery. Meanwhile, experiments of a more illusory nature include to make a man appear headless or to make a house seem as though it is full of snakes. While veterinary charms are technically medical in nature, I have made the decision to include these in this survey as they are revealing about the diversification of a practitioner’s skills and services, as well as of the concerns and anxieties of medieval patients and clients outside of their own personal physical health. The strong presence of such charms reveals the value placed on animals by the medieval client, who may have been prepared to pay for professional treatment and make recourse to magical cures for their sick livestock.

**Table 1.** Non-medical charms and *experimenta* in medieval medical manuscripts

Purpose category	Frequency
Theft	54
Love	26
Animals and vermin	26
Enemies	25
Demons, spirits and elves	23
Veterinary	18
Uncover secrets	16
Victory	15
Illusion	11
General protection	8
Win favour	8
Against inclement weather	4
Travel	3
Prevent slander	3
Fire	3
Escape captivity	3
Counter witchcraft	2
Invisibility	2

This quantitative breakdown provides us with an insight into the most common concerns and anxieties of a medieval patient or client, as well as demonstrating the particular types of issues for which they might be prepared to consult a practitioner. The most prominent utility of the texts surveyed here is—by far—to prevent or identify thieves. Invocations designed to prevent theft include the ‘God was born in Bethlehem’ charm which draws on the narrative of Jesus’s baptism in the river Jordan. Frequently, charms for theft also feature more proactive lines, including to apprehend or ‘spell-bind’ thieves.^38^ Moving away from protection or apprehension, there are a number of experiments for identifying the person who has stolen from you, including writing the suspects’ names on virgin wax or parchment, placing these inside clay balls and then floating these in water to see which ball unfurls first; drawing an eye on a wall and banging it with a stick or piece of iron with the suspects assembled before you; or the identification of the perpetrator in a dream, brought on by placing a piece of parchment with certain words or characters written on it beneath the victim’s pillow.^39^

Experiments for love, too, are common. These include practices such as placing a magnet under a woman’s head while she sleeps: if she loves you then she will embrace you, but if she gets out of the bed then she is in love with someone else.^40^ Another text recommends carrying henbane on your person when amongst women, and they will all love you.^41^ Veterinary charms also make up a considerable portion of the texts examined here. These include a simple charm to whisper in a horse’s ear, which will keep it still while it is being shod; powerful words written on bread to treat pigs with swollen throats; and spoken charms to staunch a horse’s blood, similar to those used for the same purpose in humans.^42^ Understandably the majority of concerns which are addressed by the charms and experiments in these manuscripts are problems for which there was very little other provision, thus prompting a medieval client to seek outside—and, by the requirements of the texts themselves, preternatural—assistance.

While the date range of the corpus as a whole is broad, the majority of the manuscripts originate from the period between 1350 and 1500. This reflects the huge explosion in medical and scientific writing that took place during the end of the fourteenth century and the fifteenth century.^43^ The implications of the proliferation of medical texts that ensued during this period will be discussed in more depth shortly. The earliest manuscript in the selection studied here is British Library, Sloane MS 475; the first half of the manuscript as it is currently bound was composed during the first quarter of the twelfth century, while the second half was written in the last quarter of the eleventh.^44^ Katherine Storm Hindley notes that this manuscript contains the earliest examples of Anglo-French being used in charms.^45^ Despite its chronological proximity to the Norman Conquest, Hindley finds that the manuscript’s charms bear little similarity to those from the pre-Conquest period, and instead are much closer to those of the later medieval period.^46^ Many of the charms and experiments reflect similar concerns to the later manuscripts discussed here, including for victory, against enemies and to identify a thief. One particular text, however, appears unique to Sloane MS 475 and serves a rather niche purpose: if your enemy has a lot of his wine in his cellar and you do not want him to enjoy it.^47^ Other earlier manuscripts surveyed for this study include British Library, Sloane MS 146, mostly written in the last quarter of the thirteenth century, but with a botanical glossary from the late twelfth century; British Library, Royal 12 B XII, from the thirteenth century; Oxford, Bodleian Library, MS e Mus. 219 which was written around the turn of the thirteenth century; and Paris, Bibliothèque Nationale de France, MS Nouvelles Acquisitions Latines 693 which is early fourteenth century. These codices reflect some differences to the rest of the corpus, particularly, as would be expected, in terms of language: they are almost exclusively in Latin with some instances of Anglo-Norman. The one exception is a charm against thieves in MS Nouvelles Acquisitions Latines 693, which is in English.^48^ This tallies with the limited availability of (English) vernacular medical writing prior to the middle of the fourteenth century. However, with regard to the utilities of the charms and experiments, there is little obvious distinction between these earlier manuscripts and the later ones. Prominent themes are to prevent or identify thieves, to uncover a woman’s secrets, to incite love and to defeat enemies.

## Closer Manuscript Studies

A quantitative overview of these texts is useful in order to understand the numerically significant presence of non-medical charms and experiments in otherwise medical manuscripts. However, a closer look at specific manuscripts can provide further insight and serves to contextualise the place of these practices alongside medical recipes and healing charms. The three manuscripts selected for closer examination have been chosen because they are representative of the wider corpus, but they also reflect some of the differences and diversity of the broader 48 codices. For example, British Library, Royal MS 12 B XXV is almost entirely in Latin, with a few Anglo-Norman and Middle English recipes; British Library, Additional MS 34111 is exclusively in Middle English, and British Library, Additional MS 12195 contains a mixture of Middle English and Latin texts. Each manuscript also contains a longer tract of experiments that constitute a known work, in addition to a number of individual isolated charm and experiment texts. In the case of Royal MS 12 B XXV, this is the *Liber aggregationis* of pseudo-Albertus Magnus, a tract on the virtues of herbs, stones and animal parts apocryphally attributed to the Dominican friar; Additional MS 12195 contains the *De corio serpentis* or *Twelve Experiments with Snakeskin* of Johannes Paulinus; and Additional MS 34111 has an English translation of the *Virtutes aquile*, a tract on the various medical and non-medical properties of the eagle.^49^ These three case studies, therefore, provide us with an insight into some of the different tracts and texts that were in circulation among medical practitioners at the time.

## London, British Library, Additional MS 34111

Dated to the second quarter of the fifteenth century, Additional MS 34111 is a large compendium containing 238 folios of medical tracts, treatises and recipes.^50^ The manuscript cites well-known authorities, such as Galen and Hippocrates, as well as less-easily identified figures such as ‘Rusticus’, Parisius, Abbot of St. Marks and ‘Cophon the Leche’.^51^ Almost all published scholarship on this manuscript, including the British Library catalogue itself, cites references to a Master William (fols. 114 and 169) and to Master William Somers (fol. 174) in order to suggest that the manuscript must have been compiled by someone of this name, or under his direction.^52^ Lanfranc mentions a Maister William Someris in the *Science of Cirurgie* as having made a resin ointment, while Talbot and Hammond list ‘William of Sumery’ as a thirteenth-century physician; many scholars go as far as to suggest that the original owner or compiler of Additional 34111 was this person.^53^ However, given that the manuscript is dated to the second quarter of the fifteenth century, this suggestion makes no sense: the manuscript itself could not have been compiled under the direction of a physician who was practicing two hundred years prior to its completion. While Additional 34111 could be based on an earlier exemplar which was in the possession of William Somers, it is more likely that he is instead one of the—albeit lesser known—authorities that the manuscript cites. Tony Hunt notes that a Latin tract of experiments attributed to a William Somers features in Cambridge, Corpus Christi MS 297, while Talbot and Hammond cite British Library, Royal MS 12 E XXIII as another extant witness.^54^ Comparison with the text in Additional 34111 suggests a close link.^55^

Though we cannot know who the original compiler or owner of Additional 34111 was, the manuscript evidence is strongly suggestive that this person was a medical practitioner. Entirely in one hand, meaning that it was planned and executed as a single volume, the manuscript is carefully and clearly indexed, to aid quick identification. While the book titles in the index are given in Latin, the tracts themselves are all in English.^56^ As well as works attributed to Galen and Hippocrates, the manuscript contains other texts such as an English version of the *Trotula*, labelled ‘Translation B’ by Monica Green and other works influenced by Salernitan authors such as the *Speculum medicorum* and a tract of experiments attributed to Cophon, the ‘Leche of Salerne’.^57^ The non-medical charms and experiments are outlined in Table 2.

**Table 2.** Non-medical charms and *experimenta* in British Library, Additional MS 34111

Purpose of charm or experiment	Folio number
To identify a thief	70v
To recover lost articles	71v
To escape captivity	71v
Make a man reveal secrets in his sleep	71v
Success in battle	72v
For the foundering of a horse	75r
To identify a thief	75r
Protect fields	168r
*Virtutes aquile* ^a^ A tract using the body parts of an eagle for different medical and non-medical purposes, including: for grace and friendship from lords and ladies; protection from man and wicked spirits; to gain riches; win the love of a man or woman; to see the future or discover things in a dream;	195r–196v

^a^See Suzanne Sheldon, ‘The Eagle: Bird of Magic and Medicine in a Middle English Translation of the Kyranides’, *Tulane Studies in English*, 1977, 22, 1–32.

The presence of rubricated *manicula* in the margins beside the charms for success in battle and the two methods to identify thieves suggests that these items were frequently consulted. The inclusion of these two practices to identify thieves, one through painting an eye on the wall and the other by inscribing names in virgin wax, along with a third charm for the loss or perhaps theft of items, betrays a particularly prevalent anxiety of the time.^58^ It is tempting to speculate that the charm for success in battle was regularly consulted as the manuscript was composed as the Hundred Years War drew to a close, and civil war in England began. The astrologer and medical practitioner Richard Trewythian, who was practicing during this period, performed a number of consultations to address anxieties and provide answers about upcoming battles.^59^

The *Virtutes aquile* finds its origins in the Greek *Kyranides*, a text that provides medical recipes that use the body parts of animals, birds and fish.^60^ Suzanne Sheldon, who edits the text, claims that the tract in Additional 34111 is ‘strangely out of place in a compendium of medical luminaries … which have some claim to medical validity’ and asserts that it derives from the ‘superstitious practices of folk medicine’.^61^ This assertion does not take into account the other charms and experiments in the manuscript, nor does it recognise that these types of tracts—as we shall see—are common in other medieval medical manuscripts, and clearly reflect complementary interests.

## London, British Library, Royal MS 12 B XXV

A fourteenth-century manuscript, chiefly in Latin, the 1921 catalogue notes that Royal MS 12 B XXV contains “Prayers and charms against various evils, in *Latin*, *French*, and *English*.”^62^ Comprising 284 folios, the manuscript is in just one hand, though a later, fifteenth-century hand has added three charms for lust, fire and fever on the final folio. The contents of the codex are primarily medical, and previous scholarship has contended that it would likely have been used by either a doctor or less formally trained healer.^63^ However, a number of entries also demonstrate an interest in experimental science: there is a passage on how to make Greek fire, fireworks, rockets and burning glass.^64^ This may well reflect the personal or intellectual interests of the original compiler, rather than an intention to use these texts in a professional context.

In addition to these scientific experiments, the manuscript also includes tracts on urine (fols. 9r–15v), astrology (fols. 254v–263v) and a number of passages detailing how to make medical compounds (e.g. fols. 68r–75v). Like Additional 34111, there is a diverse selection of non-medical charms and experiments integrated with the medical recipes, outlined in Table 3.

**Table 3.** Non-medical charms and *experimenta* in London, British Library, Royal MS 12 B XXV

Purpose of charm or experiment	Folio number
Against thunder	60r
For sick cattle	62r
To capture snakes	62v–63r
For pigs with swollen throats	63r
For sick pigs	63r
Against enemies	63r-v
To identify a thief	63v
For a dog that won’t bark	64v
To capture snakes	65r
To learn a woman’s secrets	65v
To interrogate a woman in her sleep	65v
Experiments of pseudo-Albertus Magnus^a^ Includes certain uses of herbs, animal parts and birds, including to detect adultery, to overcome enemies, prevent slander, identify a thief, protect your money in the marketplace and more.	248r–251r
To remove mice and rats from the house	253r
Against thieves	253v
To extinguish lust	283v
For fire	283v

^a^For a full insight into these texts and their legacy see Isabelle Draelants, *Le Liber de virtutibus herbarum, lapidum et animalium (Liber aggregationis): un texte à succès attribué à Albert le Grand*, Micrologus’ library, 22 (Firenze: SISMEL, edizioni del Galluzzo, 2007); for a closer look at the authorship of these tracts see Lynn Thorndike, ‘Further Consideration of the *Experimenta*, *Speculum Astronomiae*, and *De* S*ecretis Mulierum* Ascribed to Albertus Magnus’, *Speculum*, 1955, 30, 413–43.

While these texts coalesce around two particular sections of the manuscript, they are not discrete groupings, independent from the other material. Instead, they are interspersed with standard medical recipes such as treatment for dog bites and eye problems, and medical charms, including to staunch blood and to find out whether a sick person will live or die. The inclusion of a number of veterinary charms here adds an interesting dynamic. The consequences of sick livestock could be financially devastating: charms to heal animals not only demonstrate the high value placed on cattle but also that medical practitioners sometimes made provision for their care.

While many of the charms and experiments in this manuscript reflect similar concerns to the others examined as part of this study, some specific aspects of the texts themselves are—to my knowledge—not found in other English manuscripts from the period. For example, one of the experiments to uncover a woman’s secrets—a purpose that occurs regularly in this corpus, usually requiring the practitioner to write certain characters on virgin wax or parchment and place it beneath the woman’s head while she sleeps—here specifies that the words must be written in the blood of a bat. Others, such as an experiment—with instructions in Anglo-Norman—to identify a thief by writing certain characters on pieces of bread and feeding them to the suspect, have antecedents in earlier manuscripts with Anglo-Norman texts, demonstrating the continued use of this language in the circulation of certain texts or recipes.^65^

## London, British Library, Additional MS 12195

Additional 12195 is a late-fifteenth-century manuscript that falls into four distinct sections, three of which have evidence, both in terms of provenance information and linguistic features, of a close tie with Norfolk.^66^ The first three sections contain a myriad of texts, including specimen forms of testaments, banns, and other deeds, grammatical tracts, liturgical notes and services for certain feasts and festivals. The final section of the manuscript is a collection of medical recipes and *experimenta* and shows evidence of having circulated independently prior to being bound with the other parts.^67^ In light of this, and the fact that David Thomson, in his in-depth description of the codex, notes that it is unclear when the four sections were bound together but that it may have been as late as 1770, for the purposes of this study I consider the fourth and final section independently from the remainder.^68^

This fourth section of the manuscript (now fols. 122r–190v) contains a number of medical tracts in English, including a treatise on natural science and astronomy, the characters of people born under different signs of the zodiac, medical recipes including a textual charm for childbirth and ‘to make a watyr that good king Edward usyd’—a recipe which instructs the maker to steep herbs including fennel, vervain, celandine and betony in white wine, a child’s urine and breast milk, to treat ‘alle evyl in þe eyne’. The manuscript also contains a treatise on childbirth entitled *The Knowing of a Woman’s Kind in Childing,* a vernacular version of part of the *Trotula*, labelled ‘Translation A’ by Green.^69^ The non-medical charms and experiments are as outlined in Table 4.

**Table 4.** Non-medical charms and *experimenta* in London, British Library, Additional MS 12195

Purpose of charm or experiment	Folio number
The *De corio serpentis* of Johannes Paulinus: 12 experiments with snakeskin^a^ Includes experiments to tell friends from enemies, to make enemies flee, to be heard well during council, to win a dispute, to recover stolen objects, reveal secrets and win love and favour	122r–124r
To make the house look as though it is full of snakes	124r
To make birds fall down as though dead	124r
To find green adders	124r-v
To learn anything you want to know in a dream	124v
To become invisible	124v
To make geese turn on the spot	125r
To catch birds with your hands	125v
To make things look as though they are made of gold	126r
To have anything you ask for from a lord or lady	126v
To make a woman love you	126v
To put out a fire	136v
Conquer enemies	149r
Prove a woman’s virginity	149r
Identify a thief	149v

^a^William Eamon, ‘Medieval Wonder Drugs’.

There are clearly a number of practices here of a more illusory nature, perhaps, like the experiments to make Greek fire in Royal 12 B XXV, reflecting the personal interests of the compiler. However, a number of the other experiments reflect social concerns, particularly those that might have preoccupied a higher-status individual, providing an insight into the type of client who may have contracted a practitioner to aid with problems that went beyond the medical.^70^ This is something that will be considered in more depth shortly.

There is little evidence of interaction with the texts. A small number of *nota* have been added in a contemporary hand in the margins, as well as annotations ‘for fyre’ beside the Latin charm for fire, and ‘for feveres’ beside a healing charm, demonstrating some engagement with the texts after the manuscript’s compilation. The *De corio serpentis* is in English: this is rare, as the text usually occurs in Latin.^71^ The focus of Additional 12195 on vernacular texts, with both this tract and the recension of the *Trotula* being copied in translation rather than Latin, does suggest a compiler with a preference for reading English texts, although we cannot rule out that this may be reflective instead of the scribe’s access to specific exempla. However, there are a number of Latin texts, including the experiment to become invisible and the charm to put out a fire, implying at least some level of Latin literacy.

Alexandra Barratt uses certain textual and codicological features to argue that the copy of *The Knowing of Woman’s Kind in Childing* in this manuscript was used by women. She cites several clumsy scribal mistakes and misspellings that have been made in the copying of a number of medical and gynaecological terms as evidence that even the redactor, not just the reader, may have been female, while she describes this portion of the manuscript as featuring ‘extensive rubrication’, which may have made it more easy to consult and navigate by ‘those known to be relatively inexperienced with books’.^72^ However, I would contend that neither of these observations constitutes evidence of a female readership. The ‘extensive’ rubrication described by Barratt, principally marking the beginning of new passages and underlining key words, is not unusual in manuscripts of medical material, and served as an organisational and navigational aid for practitioners who might need to find and consult material in haste.^73^ The scribal errors are not sufficient evidence for a female scribe or reader, and may in fact reflect the quality of the exemplar used for copying, or otherwise simply signify the redactor’s lack of learning or familiarity with gynaecological texts, something which is not necessarily an indicator of their gender.

## A Text-based Magico-Medical Practice

This closer examination of three manuscripts shows that, while there is certainly crossover in terms of contents, no two practices offered by a physician were the same. However, we can nevertheless identify some key characteristics of the magico-medical practitioner which can be broadly applied. First, the manuscripts studied here were likely all owned by men, and thus reflect the interests and offerings of male practitioners. While the inclusion of *Trotula* texts in two of the codices examined above makes it tempting to consider the possibility of female readership and, as mentioned above in relation to Additional MS 12195, has prompted arguments in favour of this suggestion, Monica Green’s extensive study of *Trotula* texts finds that ‘most written knowledge about women’s bodies is to be found in texts composed *by* male physicians and surgeons, *for* male physicians and surgeons’.^74^ Low female literacy rates make it unlikely that these codices were owned by women, and the heavy presence of Latin texts and tracts in this corpus as a whole almost certainly excludes the possibility that these manuscripts reflect the practice of female physicians.^75^ While there is also a strong presence of vernacular literature in these manuscripts, reflecting the increased availability of medical texts in English, the frequency with which Latin occurs also suggests that their owners had attained a reasonable level of education, while it is axiomatic that the ability to commission or purchase such a codex precludes ownership by the poorest or humblest strata of society. Beyond these similarities, the diversity of materials collected in these codices was likely influenced by a number of factors, including personal inclination, client demand, economic and social incentives and access to—and transmission of—medical knowledge, including available sources for copying. For the remainder of this essay, I will consider some of the factors that may have led a practitioner to diversify their practice and include services that went beyond the medical.

The last quarter of the fourteenth century witnessed the beginning of a translation movement in England. Until this point, Latin had been the primary language of scientific and medical discourse; however, by the end of the fifteenth century we find that a broad range of university treatises had become available in English.^76^ Coupled with this increased availability of vernacular texts was a rise in lay literacy. Though not dramatic—literacy rates in the fifteenth century have been estimated to be somewhere between 5 and 15 per cent, although higher in urban areas—this created a new readership that might have had a vested interest in these newly translated texts.^77^ This bivalent increase in access to medical writing gave rise to a broadening in the domain of what Monica Green calls ‘literate medicine’, a category which, while encompassing learned medicine, expands to include any written material, such as recipes noted in a commonplace book.^78^

This broadening domain of literate medicine and the increased potential for readership that accompanied it, likely influenced the process of book production. In an examination of the format and design of a number of, mainly fifteenth century, vernacular medical manuscripts, Rossell Hope Robbins suggests that they may have been compiled in commercial scriptoria for speculative sale.^79^ While this notion of commercial scriptoria has since been largely disproved, nevertheless there are groupings of manuscripts with similarities that may point towards an individual or publisher specialising in scientific and medical texts.^80^ For example, Linda Ehrsam Voigts identifies the ‘Sloane Group’, a series of fifteenth-century medical and scientific codices in the British Library’s Sloane collection, which possess a number of similarities in terms of paper sources, texts and *mise-en-page*, suggesting that their production was overseen by either an individual or an organisation specialising in scientific and medical texts.^81^ While codices like Additional 34111 and Royal B 12 XXV described above are in one hand, and therefore likely the product of a specific commission, other manuscripts surveyed for this study may have been slowly built up over time as specific texts and tracts were purchased and bound with the rest. British Library, Sloane MS 121, for example, is compiled of a number of individual booklets that are self-contained within one or more quires.^82^ Two of these booklets, one containing a treatise on urines and the other, one of the *Trotula* texts, show distinct similarities with the same tracts found in the commonplace book of Humphrey Newton.^83^ In his study of Newton’s commonplace book, Ralph Hanna suggests that these two tracts formed the core of the manuscript around which the other texts were collected, and may have been a bespoke commission, perhaps from a scribe who regularly copied medical texts.^84^

The latter end of the medieval period, however, did not just experience an increase in vernacular medical writing: it also bore witness to the changing interests of manuscript readers and compilers. While theoretical tracts were still circulating, there was a demonstrable increase in appetite for knowledge that could yield practical results, such as medical recipes and remedies that had been proven to work by past experience.^85^ The charms and *experimenta* discussed above fall into this category, and their inclusion in medical manuscripts from the late-fourteenth century onwards reflects this interest in experiential medicine. Lea Olsan’s assessment of the corpus of medical charms in the ‘Leechbook’, a tract of medical recipes which includes a number of healing charms, supports the argument for increased textual transmission of practical knowledge with specific reference to charms and *experimenta*. Olsan demonstrates that there is a core set of charms copied into the majority of surviving manuscripts containing the Leechbook; not only do they serve a set list of functions, they also share the same motifs and almost identical wording, suggesting textual rather than oral transmission. The most obvious of these is the charm against thieves which uses the ‘God was born in Bethlehem’ motif mentioned above: this text occurs in ten of the manuscripts I have studied, of which eight use almost identical phrasing.^86^

The above observations demonstrate that there was an increased opportunity for participation in medical practice for those outside of the spheres of university or clerical orders, grounded in broadening access to textual medicine, as well as an increase in the availability of shorter, more practical medical treatises, charms and experiments. Having established that medical literature was more widely available for practitioners to commission or purchase, other motivating factors for broadening medical practice to include non-medical components remain to be explored.

After the Black Death wiped out a significant proportion of Europe’s population, the number of physicians did not drop.^87^ This suggests that the appetite for, and capacity for patients to purchase, medical assistance had significantly risen, but it also meant that there was now a far higher number of physicians or medical practitioners *per capita*. We know, also, that there was an increasing prominence of non-university-trained physicians in England by the early fifteenth century, evidenced by a petition submitted by graduate doctors in 1421 to prevent those without a university qualification from practising medicine.^88^ This was passed as law in 1428 but it proved impossible to enforce; however, such an attempt to suppress the practices of lesser-qualified physicians shows increased competition amongst those with different levels of learning and education. Practitioners would therefore have been forced to define their services and delineate what set them apart from their competitors: access to superior knowledge was one particular commodity that could prove lucrative. Thomas Fayreford notes in his manuscript that he was paid money by barbers to share a tip about using the skin of a green frog to remove teeth.^89^ We can surmise that practitioners were prepared to invest money to acquire a skill or service that would attract clients. A similar attitude towards the acquisition of non-medical skills may well have been a contributing factor in the evolution of the magico-medical practitioner.

One of the most lucrative non-medical skills was theft prevention or detection; thus it makes sense that this is the most frequent addition to the medical recipes in the manuscripts studied here.^90^ Identifying thieves was something for which medieval society made little provision, yet the loss of even simple items such as clothing, much less economically vital possessions such as livestock, could be devastating.^91^ Recourse to magical means to protect oneself from, or detect, theft must have appealed to many. The identification of thieves was an integral part of the practice of cunning folk, but there is evidence that it was performed by other types of practitioners too.^92^ For example, the manuscript of medical practitioner, astrologer and moneylender Richard Trewythian shows that this was one of his most frequently employed services.^93^ However, the use of astrology for theft detection was just one method among a variety of options for tackling this problem, something which is strongly evidenced by the assortment of charms and other magical techniques relating to theft that appear in the manuscripts surveyed here. The popularity of practices to tackle theft suggests that the inclusion of supplementary services was a way for medical practitioners to tap into a new market that catered to concerns beyond the physical body, thus expanding their potential to generate income.

New skills are only lucrative if, like practices to tackle theft, they respond to a particularly prevalent concern. In this way, we can surmise that in many cases, the diversification of a practitioner’s offering would have been motivated by client demand. Studies have shown that the reputation of the practitioner, and trust between practitioner and client, was of paramount importance, and that these factors influenced patients when choosing who to consult for assistance with medical—and other—problems.^94^ Trust, and a continued relationship between client and practitioner, would likely have led to the discussion of issues beyond the medical; consultations may have moved on to centre more intimate concerns. For example, almost all of the manuscripts surveyed include at least one recipe or charm for childbirth, most commonly instructions to create a textual amulet which is then fastened to the labouring woman’s thigh or stomach.^95^ But from the manuscript evidence we can surmise that patients did not only seek assistance at the moment of delivery: there are also a large number of *experimenta* for fertility and conception. British Library, Additional MS 33996, for example, explains how to make an amulet to wear on the body during intercourse. The instructions suggest that the efficacy of the amulet can be tested by suspending it on a tree to make it bear fruit.^96^

These recipes reveal the practitioner as an influencing presence during both conception and delivery. Tabitha Stanmore cites known cases of women consulting practitioners for assistance with fertility, before contracting them to provide other, extra-medical services, such as love magic and prognostication.^97^ This, therefore, may explain the presence of a number of different charms and experiments to incite love or lust, to provoke marital harmony or discord, and to detect adultery. Stockholm, Royal Library, MS X.90 contains three experiments in this domain: one for love, youth and beauty, one to guarantee a happy marriage and another to detect virginity.^98^ Similarly, Additional 12195 includes an experiment to make a woman love you.^99^ Conversely, Royal 12 B XXV includes a charm to extinguish lust, as well as two experiments to learn a woman’s secrets.^100^

These practices offer us a fascinating insight into the state of medieval relationships and their key concerns and anxieties. In reference to the use of medical charms in healing, Olsan asserts that ‘when the doctor or other healer is confronted with an individual patient’s distress, then one or more therapies from a range of praxes related in various ways to the dominant discourse may be applied’.^101^ We can extrapolate this and apply it to the discussion of non-medical concerns within a patient–practitioner consultation, and suggest that, when met with the anxieties of a client, the practitioner was motivated to find a suitable solution.

I have already suggested that the possession of certain knowledge might be economically advantageous and could set a practitioner apart from the competition. However, access to a specific, more occult, knowledge, might be seen to lend the practitioner further prestige. In an examination of manuscripts of ritual magic, Frank Klaassen argues that the possession of occult knowledge contributed to the construct of masculinity, and that it presented the practitioner as ‘intelligent, materially successful, controlled, and bold’.^102^ While the non-medical charms and experiments that appear in the manuscripts surveyed do not conjure or compel demons to bring about results, unlike the ritual magic examined by Klaassen, I would argue that the possession of—sometimes esoteric—knowledge would still contribute to the status of the practitioner. This is particularly the case for items that involve uncovering secrets, identifying thieves or prophesying future events through occult means. Writing esoteric characters on parchment for a victim of theft to place under their pillow, or using animal parts to reveal someone’s secrets during sleep, demonstrated that the practitioner possessed the means to access information not available to ordinary people.

This occult knowledge, when combined with access to much of the corpus of practical medical knowledge that circulated among university-trained physicians, gave this particular type of magico-medical practitioner a specific space within the hierarchy of the medical profession. In her study of the manuscript of astrologer Richard Trewythian, Sophie Page suggests that his status somewhere between that of humble diviner and court astrologer offered clients a convenient middle-ground, an affordable and accessible practitioner whose knowledge was nevertheless grounded in textual learning, and who could perhaps be seen to offer a more ‘sophisticated’ practice.^103^ We can make similar interpretations when exploring the range of methods available for theft detection in the manuscripts studied. In particular, there are some methods that were known to have been in use at the time, but which are notable by their absence from the sources examined here. These include the ‘sieve and shears’ and ‘book and key’ methods, in which the latter object is placed on or into the former, and is supposed to turn around at the mention of the guilty party.^104^ Such methods may have been more accessible to practitioners who were unlearned and unlettered, while methods such as using clay balls or parchment under the pillow required—at least some—literacy on the part of the practitioner.^105^ Practitioners who drew their techniques from a textual source—and on occasion actually employed their own literacy as a means to enact theft detection—while also boasting access to a written corpus of medical and occult knowledge, may well have provided a more attractive and trustworthy option for a client seeking a more sophisticated practice.

While records of consultations in manuscripts such as those owned by Richard Trewythian or Thomas Fayreford reveal that these practitioners treated many different levels of society, Tabitha Stanmore’s recent study suggests that the *type* of services requested by clients varied slightly according to the client’s status. For example, love magic, including to gain favour, as well as magic for success in gambling, and more elaborate and expensive magic was favoured by the upper classes.^106^ Thus we might interpret the presence of charms and experiments for these purposes in the manuscripts surveyed, as reflective of both the practitioner’s desire for status, as well as an attempt to appeal to a higher-status clientele.

## Conclusion

An investigation of the non-medical charms and experiments in the 48 manuscripts selected for study demonstrates that, towards the end of the medieval period there were numerous practitioners who supplemented their medical practice with other services not exclusively related to healing. The manuscript evidence, including the strong presence of vernacular texts and practical material, suggests that the evolution of this type of practitioner was likely facilitated by the explosion in vernacular medical writing, as well as the increasing interest in experiential medicine. Meanwhile, considering economic factors such as the competitive nature of medical practice and the drive to address the client’s needs, helps us to hypothesise as to why a practitioner may have expanded their services to provide solutions to problems that stood outside of the domain of healing. While the type of practitioner revealed by these manuscripts share many commonalities with others operating within the medical profession, such as the leech, educated but not university-qualified physician like Thomas Fayreford, or the master surgeon John Arderne, as well as magico-medical practitioners such as cunning folk, their combination of text-based authoritative medical knowledge with magical solutions to other problems gave them a niche in what was otherwise a very crowded marketplace.

## Footnotes

1 London, British Library, Sloane MS 56, fol. 79r.

2 However, two charms against thieves have been added to the final folio of the manuscript by a later reader, see Lea Olsan, ‘Charms and Prayers in Medieval Medical Theory and Practice’, *Social History of Medicine*, 2003, 16.3, 343–66, 346, note 15, though note that the reference here should be to Sloane MS 56 as Olsan indicates in Table 1 on 365; for transcriptions of these two additional charms see Suzanne Sheldon, *Middle English and Latin Charms, Amulets, and Talismans from Vernacular Manuscripts* (unpublished PhD thesis, University of Tulane, 1978), 133, 135.

3 Peregrine Horden provides a useful summary of the negative reception of such texts in ‘What’s Wrong with Early Medieval Medicine?’, *Social History of Medicine*, 2011, 24, 5–25.

4 Olsan, ‘Charms and Prayers’.

5 See, for example, Richard Kieckhefer, *Magic in the Middle Ages*, 2nd edn (Cambridge: Cambridge University Press, 2014), 8–17 and ‘Rethinking How to Define Magic’, in Sophie Page and Catherine Rider, eds, *The Routledge History of Medieval Magic* (London: Routledge, 2019), 15–25.

6 Kieckhefer, ‘Rethinking How to Define Magic’, 16.

7
*Ibid*., 16.

8 Lea Olsan, ‘Charms in Medieval Memory’, in Jonathan Roper, ed, *Charms and Charming in Europe* (Basingstoke: Palgrave Macmillan, 2004), 59–88, 60.

9 Peter Murray Jones, ‘*Experimenta*, Compilation and Construction in Two Medieval Books’, *Poetica*, 91/92 (2019), 61–80 (69–70); see also Michael McVaugh, ‘The Experiments of Arnald of Villanova’, *Journal of Medieval and Renaissance Studies*, 1971, 1.1, 107–18.

10 Brian P. Copenhaver, ‘A Tale of Two Fishes: Magical Objects in Natural History from Antiquity Through the Scientific Revolution’, *Journal of the History of Ideas*, 1991, 52.3, 373–98.

11 Olsan, ‘Charms and Prayers’, 348.

12 Lea Olsan, ‘The Marginality of Charms in Medieval England’, in James Alexander Kapaló, ed, *The Power of Words: Studies on Charms and Charming in Europe* (Budapest: Central European University Press, 2013), 135–64, 153.

13 T. M. Smallwood, ‘The Transmission of Charms in English, Medieval and Modern’, in *Charms and Charming in Europe*, 11–31; Olsan, ‘Charms in Medieval Memory’, 63.

14 As well as the works cited by Olsan above, see Peter Murray Jones and Lea Olsan, ‘Performative Rituals for Conception and Childbirth in England, 900–1500’, *Bulletin of the History of Medicine*, 2015, 89, 406–33; Peter Murray Jones, ‘Harley MS 2558: A Fifteenth-Century Medical Commonplace Book’, in Margaret R. Schleissner, ed, *Manuscript Sources of Medieval Medicine: A Book of Essays* (New York: Garland, 1995), 35–54; Catherine Rider, ‘Medical Magic and the Church in Thirteenth-Century England’, *Social History of Medicine*, 2011, 24, 92–107.

15 Eamon Duffy, *The Stripping of the Altars: Traditional Religion in England; c. 1400 - c. 1580*, 2nd edn (New Haven: Yale Univ. Press, 2005), 266–87; Kieckhefer, *Magic in the Middle Ages*, 56–94.

16 Owen Davies, *Cunning-Folk: Popular Magic in English History* (London: Hambledon and London, 2003).

17
*Ibid*., 15; see also Keith Thomas, *Religion and the Decline of Magic: Studies in Popular Beliefs in Sixteenth- and Seventeenth-Century England*, Penguin Religion, Repr (London: Penguin Books, 1991) and Alan Macfarlane, *Witchcraft in Tudor and Stuart England: A Regional and Comparative Study*, Repr (London: Routledge, 1999).

18 Tabitha Stanmore, *Love Spells and Lost Treasure: Service Magic in England from the Later Middle Ages to the Early Modern Era* (Cambridge: Cambridge University Press, 2022).

19
*Ibid*., 11.

20
*Ibid*., 227.

21 Faye Marie Getz, *Medicine in the English Middle Ages* (Princeton, NJ: Princeton University Press, 1998), 15.

22 Julie Orlemanski, *Symptomatic Subjects: Bodies, Medicine, and Causation in the Literature of Late Medieval England*, Alembics (Philadelphia: University of Pennsylvania press, 2019), 21, 50.

23
*Ibid*., 21; Getz, *Medicine in the English Middle Ages*, 17, 65.

24 Orlemanski, *Symptomatic Subjects*, 22.

25
*Ibid*., 22–23.

26 Getz, *Medicine in the English Middle Ages*, 6.

27 London, British Library, Harley MS 1735.

28 James K. Mustain, ‘A Rural Medical Practitioner in Fifteenth-Century England’, *Bulletin of the History of Medicine*, 1972, 46, 469–76, 472.

29 London, British Library, Harley MS 2558.

30 Peter Murray Jones, ‘Witnesses to Medieval Medical Practice in the Harley Collection’, *British Library Journal*, 2008, 8, 1–13, 3, and ‘Thomas Fayreford: An English Fifteenth-Century Medical Practitioner’, in Andrew Cunningham, ed, *Medicine from the Black Death to the French Disease* (Aldershot: Ashgate, 1998), 156–83.

31 London, British Library, Sloane MS 428.

32 For further discussion of this manuscript, see Sophie Page, ‘Richard Trewythian and the Uses of Astrology in Late Medieval England’, *Journal of the Warburg and Courtauld Institutes*, 2001, 64, 193–228.

33 Jones, ‘Witnesses to Medieval Medical Practice’; Tig Lang, ‘Medical Recipes from the Yorkist Court’, *The Ricardian*, 2010, 20, 94–102.

34 Linda Ehrsam Voigts, ‘Fifteenth-Century English Banns Advertising the Services of an Itinerant Doctor’, in Florence Eliza Glaze and Brian Nance, eds, *Between Text and Patient: The Medical Enterprise in Medieval & Early Modern Europe*, Micrologus’ Library, 39 (Firenze: SISMEL, Edizioni del Galluzzo, 2011), 245–79.

35 See Appendix 1 for a full list of manuscripts surveyed; these have been sourced both from existing databases or surveys of magic in medieval manuscripts, such as Sheldon, *Middle English and Latin Charms*; Laura Mitchell, ‘Cultural Uses of Magic in Fifteenth-Century England’ (unpublished PhD thesis, University of Toronto, 2012); George R. Keiser, *A Manual of the Writings in Middle English, 1050-1500*, 10 vols, Albert E. Hartung and John Edwin Wells, eds (New Haven: Connecticut Academy of Arts and Sciences, 1998); Lynn Thorndike, *A History of Magic and Experimental Science*, 8 vols (New York: Macmillan, 1923); and Lynn Thorndike, *A Catalogue of Incipits of Medieval Scientific Writings in Latin* (Cambridge, MA: The Mediaeval Academy of America, 1963), and the online edition of Linda Ehrsam Voigts and Patricia Deery Kurtz, *Scientific and Medical Writings in Old and Middle English: An Electronic Reference* (Ann Arbor: University of Michigan Press, 2000), as well as through my own archival investigations.

36 A good example of a household manuscript containing a vast array of non-medical charms and experiments is Oxford, Bodleian Library, MS Digby 86, see Marjorie Harrington, ‘Science, Medicine, Prognostication: MS Digby 86 as a Household Almanac’, in Susanna Fein, ed, *Interpreting MS Digby 86: A Trilingual Book from Thirteenth-Century Worcestershire* (Woodbridge: Boydell & Brewer, 2019), 55–72 and Jennifer Jahner, ‘Literary Therapeutics: Experimental Knowledge in MS Digby 86’, in *Interpreting MS Digby 86*, 73–86.

37 This figure is based on a preliminary study of each manuscript, but would likely grow subsequent to further scrutiny.

38 Chiara Benati, ‘Painted Eyes, Magical Sieves and Carved Runes: Charms for Catching and Punishing Thieves in the Medieval and Early Modern Germanic Tradition’, in Albrecht Classen, ed, *Magic and Magicians in the Middle Ages and the Early Modern Time: The Occult in Pre-Modern Sciences, Medicine, Literature, Religion, and Astrology*, Fundamentals of Medieval and Early Modern Culture, 20 vols (Berlin: De Gruyter, 2017), 149–218, 162.

39 An experiment with clay balls can be found in London, British Library, Sloane MS 121, fols. 36v–37r; the ‘Eye of Abraham’ experiment is in London, British Library, Additional MS 34111, fol. 75r; the dream vision charm is in London, British Library, Sloane MS 963, fols. 22v–23r.

40 London, British Library, Sloane MS 146, fols. 63v–64r; see Tony Hunt, *Popular Medicine in Thirteenth-Century England: Introduction and Texts* (Cambridge: D.S. Brewer, 1990), 290.

41 London, British Library, Sloane MS 2457, fol. 4v.

42 Cambridge University Library, MS Dd. Iv. 44, fol. 25v; London, British Library, Royal MS 12 B XXV, fol. 63r; and London, British Library, Sloane MS 962, fol. 135v.

43 See Orlemanski, *Symptomatic Bodies*, 1–2; Linda Ehrsam Voigts, ‘Scientific and Medical Books’, in Jeremy Griffiths and Derek Albert Pearsall, eds, *Book Production and Publishing in Britain, 1375-1475*, Cambridge Studies in Publishing and Printing History (Cambridge: Cambridge University Press, 1989), 345–402.

44 Katherine Storm Hindley, *Textual Magic: Charms and Written Amulets in Medieval England* (Chicago: The University of Chicago Press, 2023), 148.

45
*Ibid*., 172.

46
*Ibid*., 147.

47 See Lea Olsan, ‘Writing on the Hand in Ink: A Late Medieval Innovation in Fever Charms in England’, *Incantatio. An International Journal on Charms, Charmers and Charming*, 2018, 7, 9–45, 43.

48 For a more in-depth breakdown of language use in charms during the Anglo-Norman period see Hindley, *Textual Magic*, 151–72.

49 On the *Liber aggregationis* see Isabelle Draelants, *Le Liber de virtutibus herbarum, lapidum et animalium (Liber aggregationis): un texte à succès attribué à Albert le Grand*, Micrologus’ library, 22 (Firenze: SISMEL, edizioni del Galluzzo, 2007); on the *De corio serpentis* see William Eamon, ‘Medieval Wonder Drugs: Two Thirteenth Century Snake Tracts’, 2014, 1–16; on the *Virtutes aquile* see Suzanne Sheldon, ‘The Eagle: Bird of Magic and Medicine in a Middle English Translation of the Kyranides’, *Tulane Studies in English*, 1977, 22, 1–32.

50 The British Library catalogue merely suggests fifteenth century, *Catalogue of Additions to the Manuscripts in the British Museum in the years MDCCLXXXVIII-MDCCCXCIII* (London: 1894 repr. 1969), 198–200, while Suzanne Sheldon dates the manuscript to between 1420 and 1450 in her description in *Middle English and Latin Charms*, 73–74, and Tony Hunt to the second quarter of the fifteenth century in *Anglo-Norman Medicine: Volume II Shorter Treatises* (Woodbridge: D. S. Brewer, 1997), 10.

51 Lynn Thorndike suggests that the Rusticus referred to here might be Rusticus Elpidus, physician to Theodoric, king of the Ostrogoths in *A History of Magic and Experimental Science*, IV, 803; on Cophon see Patrick Fordyn, *The ‘Experimentes of Cophon, the Leche of Salerne’* (Brussels: UFSAL, 1983).

52
*Catalogue of Additions to the Manuscripts in the British Museum* 198; Sheldon, *Middle English and Latin Charms*, 73–74; Monica H. Green, ‘Obstetrical and Gynecological Texts in Middle English’, *Studies in the Age of Chaucer*, 1992, 14, 53–88, 60; Fordyn, *The ‘Experimentes of Cophon, the Leche of Salerne’*, 9; Stephen Stallcup, ‘The “Eye of Abraham” Charm for Thieves: Versions in Middle and Early Modern English’, *Magic, Ritual, and Witchcraft*, 2015, 10, 23–40, 36; W. L. Braekman, *Studies on Alchemy, Diet, Medecine [Sic] and Prognostication in Middle English* (Brussels: UFSAL, 1986), 115.

53 Lanfranc’s reference to William Somers can be found in Oxford, Bodleian Library, Ashmole MS 1396, fol. 262r; C. H. Talbot and E. A. Hammond, *The Medical Practitioners in Medieval England: A Biographical Register* (London: Wellcome Historical Medical Library, 1965), 416.

54 Hunt, *Anglo-Norman Medicine*, 10; Talbot and Hammond, *The Medical Practitioners in Medieval England*, 416.

55 Both of the Latin tracts begin with a recipe for ‘tinea’ or ‘tyneam’, a disease of the skin of the head which is characterised by scabs; this recipe is absent in the Additional manuscript, which begins with a brevet or textual charm for epilepsy. This brevet is the second entry in the two Latin tracts. In all three manuscripts, the recipes for epilepsy are followed by an entry for redness of eye, using ‘*cortex bugiae*’ or ‘bugie’, the bark of barberry root and for weeping eyes, using lead powder.

56 The verse introduction to the *Speculum medicorum*, a composite therapeutic manual that draws on a number of Salernitan authors, is in Latin, followed by a translation, see Klaus-Dietrich Fischer, ‘A Mirror for Deaf Ears? A Medieval Mystery’, *Electronic British Library Journal*, 2008, 1–16, 10.

57 Green, ‘Obstetrical and Gynecological Texts’, 68; Fischer, ‘A Mirror for Deaf Ears’, 10; Fordyn, *The ‘Experimentes of Cophon, the Leche of Salerne’*.

58 The ‘Eye of Abraham’ experiment to identify a thief has been edited in three separate places, see Stallcup, ‘The “Eye of Abraham”’; Braekman, *Studies*; and Sheldon, *Middle English and Latin Charms*.

59 Page, ‘Richard Trewythian’, 202.

60 Sheldon, ‘The Eagle: Bird of Magic and Medicine’.

61
*Ibid*., 4.

62 George F. Warner and Julius P. Gilson, *Catalogue of Western Manuscripts in the Old Royal and King’s Collections*, 4 vols (London, 1921), II, 19–22.

63 Lea Olsan makes this suggestion in her examination of some of the Latin charms in the manuscript in Lea Olsan, ‘Latin Charms in British Library, MS Royal 12.B.XXV’, *Manuscripta*, 1989, 33, 119–28, 119.

64 Fols. 245r–247r.

65 For example, a similar text can be found in the late-thirteenth century manuscript Oxford, Bodleian Library, Digby MS 86, fol. 20v; on the use of Anglo-Norman in charms see Hindley, *Textual Magic,* 151–72.

66 David Thomson, *A Descriptive Catalogue of Middle English Grammatical Texts* (New York: Garland, 1979), 193–211.

67 Namely, the numbering of each folio of this section, which is not replicated in the other three sections, as well as signs of wear and tear on the first folio of the section suggesting that it initially travelled without a binding or in a less protective wrapping.

68 Thomson, *A Descriptive Catalogue*, 211.

69 Green, ‘Obstetrical and Gynecological Texts’, 59; Alexandra Barratt, ed, *The Knowing of Woman’s Kind in Childing: A Middle English Version of Material Derived from the Trotula and Other Sources*, Medieval Women: Texts and Contexts, 4 (Turnhout: Brepols, 2001), 17–18.

70 This is something supported by Tabitha Stanmore’s investigation of the magical services required by the elite, in *Love Spells and Lost Treasure*, particularly 177–245.

71 The only other instance of this text in the vernacular that I am aware of can be found in San Marino, Huntington Library MS HM 64.

72 Barratt, *The Knowing of Woman’s Kind in Childing*, 34–37.

73 Hannah Bower, *Middle English Medical Recipes and Literary Play, 1375-1500* (Oxford: Oxford University Press, 2022), 111.

74 Monica H. Green, *Making Women’s Medicine Masculine: The Rise of Male Authority in Pre-Modern Gynaecology* (Oxford: Oxford University Press, 2008), 19.

75 Green, *Making Women’s Medicine Masculine*, 38.

76 Linda Ehrsam Voigts, ‘What’s the Word? Bilingualism in Late-Medieval England’, *Speculum*, 1996, 71, 813–26; Päivi Pahta and Irma Taavitsainen, ‘Vernacularisation of Scientific and Medical Writing in its Sociohistorical Context’, in Irma Taavitsainen and Päivi Pahta, eds, *Medical and Scientific Writing in Late Medieval English*, Studies in English Language (Cambridge: Cambridge University Press, 2004), 1–22.

77 Ralph Alan Griffiths, ed, ‘The Fourteenth and Fifteenth Centuries’, *The Short Oxford History of the British Isles* (Oxford: Oxford University Press, 2003), 18.

78 Green, *Making Women’s Medicine Masculine*, 12.

79 Rossell Hope Robbins, ‘Medical Manuscripts in Middle English’, *Speculum*, 1970, 45, 393–415, 413.

80 For updated scholarship on book production, see, for example, C. Paul Christianson, ‘Evidence for the Study of London’s Late Medieval Manuscript-Book Trade’, in *Book Production and Publishing in Britain*, 87–108 and Linne R. Mooney, ‘Vernacular Literary Manuscripts and Their Scribes’, in Alexandra Gillespie and Daniel Wakelin, eds, *The Production of Books in England 1350–1500*, Cambridge Studies in Palaeography and Codicology (Cambridge: Cambridge University Press, 2011), 192–211.

81 Linda Ehrsam Voigts, ‘The Sloane Group: Related Scientific and Medical Manuscripts of the 15th Century in the Sloane Collection’, *British Library Journal*, 1995, 16, 26–57; for a more recent and in-depth study of these manuscripts, see Alpo Honkapohja, *Alchemy, Medicine, and Commercial Book Production: A Codicological and Linguistic Study of the Voigts-Sloane Manuscript Group*, Texts and Transitions, 9 vols (Turnhout: Brepols, 2017).

82 For more on booklets in medieval manuscripts see Ralph Hanna, ‘Booklets in Medieval Manuscripts: Further Considerations’, *Studies in Bibliography*, 1986, 39, 100–111, and Voigts, ‘Scientific and Medical Books’.

83 Ralph Hanna, ‘Humphrey Newton and Bodleian Library, MS Lat. Misc. C. 66’, *Medium Ævum*, 2000, 69, 279–91, 282–83.

84 Hanna, ‘Humphrey Newton’, 283.

85 Peter Murray Jones, ‘Information and Science’, in Rosemary Horrox, ed, *Fifteenth Century Attitudes: Perceptions of Society in Late Medieval England* (Cambridge: Cambridge University Press, 1994), 97–111, 100.

86 The ‘God was born in Bethlehem’ charm against thieves occurs in 10 of the manuscripts I have studied. Manuscripts as follows: London, Wellcome Historical Medical Library MS 542, fol. 15r; London, British Library MSS: Additional 33996, fol. 113r; Harley 1600, fol. 30r-v; Sloane 468, fol. 61v; Sloane 374, fol. 48v; Sloane 962, fol. 51r; Sloane 2584, fol. 73v; Sloane 393, fol. 183v; Sloane 56, fol. 100v; Oxford, Bodleian Library, Ashmole 1447, p. 34; only Sloane 56 and Sloane 2584 use this motif in a differently worded charm.

87 Nancy G. Siraisi, *Medieval & Early Renaissance Medicine: An Introduction to Knowledge and Practice* (Chicago: University of Chicago Press, 1990), 24.

88 Justin Colson and Robert Ralley, ‘Medical Practice, Urban Politics and Patronage: The London “Commonalty” of Physicians and Surgeons of the 1420s’, *The English Historical Review*, 2015, 130.546, 1102–31.

89 Jones, ‘Harley MS 2558’, 52.

90 Catherine Rider, *Magic and Religion in Medieval England* (London: Reaktion Books, 2012), 66.

91 Emma Wilby, *Cunning Folk and Familiar Spirits: Shamanistic Visionary Traditions in Early Modern British Witchcraft and Magic* (Brighton: Sussex Academic Press, 2005), 37.

92 Davies, *Cunning-Folk*, 96.

93 Page, ‘Richard Trewythian’, 205.

94 Stanmore, *Love Spells and Lost Treasure*, 86, 90, 94, 99.

95 For example, London, Wellcome Historical Medical Library, MS 542, fol. 12.

96 Fol. 153v.

97 Stanmore, *Love Spells and Lost Treasure*, 190.

98 Found, respectively, on the following pages of the manuscript: 67, 61, 77.

99 Fol. 126v.

100 The charm to extinguish lust is on fol. 283r and the charm to learn a woman’s secrets on fol. 65v.

101 Olsan, ‘Charms and Prayers’, 363–64.

102 Frank Klaassen, ‘Learning and Masculinity in Manuscripts of Ritual Magic of the Later Middle Ages and Renaissance’, *The Sixteenth Century Journal*, 2007, 38, 49–76, 65.

103 Page, ‘Richard Trewythian’, 193.

104 Thomas, *Religion and the Decline of Magic*, 253–54.

105 This is backed up by Stanmore’s study, where she suggests that the gender divide seen between these practices is due to disparity in literacy rates between men and women, in *Love Spells and Lost Treasure*, 78.

106
*Ibid*., 203, 213, 227.

## Notes

Heather A. Taylor is a researcher in the Centre for Medieval and Early Modern Studies at the University of Kent. Her research examines ‘non-medical’ charms and *experimenta* in late-medieval manuscripts, particularly those to do with social relations. Her work explores what these reveal about the prominent concerns of the period and how they resonate with our broader understanding of social attitudes and anxieties in the Middle Ages.

## Appendix 1

The following manuscripts were surveyed for this study.

Cambridge University Library, Additional MS 9308

Cambridge University Library, MS Dd vi.29

Cambridge University Library, MS Dd. 5. 76

Cambridge University Library, MS Dd. iv. 44

Cambridge, Trinity College, MS O. 1. 13

Cambridge, Trinity College, MS O. 1. 65 (1089)

Cambridge, Trinity College, MS R. 14. 51

Cambridge, Trinity College, MS R. 14. 52

Cambridge, Gonville and Caius College, MS 190/223

London, British Library, Additional MS 12195

London, British Library, Additional MS 19674

London, British Library, Additional MS 27329

London, British Library, Additional MS 33996

London, British Library, Additional MS 34111

London, British Library, Arundel MS 42

London, British Library, Harley MS 3

London, British Library, Harley MS 1600

London, British Library, Harley MS 2369

London, British Library, Harley MS 2558

London, British Library, Royal MS 12 B XII

London, British Library, Royal MS 12 B XXV

London, British Library, Royal MS 12 D XII

London, British Library, Sloane MS 56

London, British Library, Sloane MS 121

London, British Library, Sloane MS 146

London, British Library, Sloane MS 374

London, British Library, Sloane MS 393

London, British Library, Sloane MS 468

London, British Library, Sloane MS 475

London, British Library, Sloane MS 962

London, British Library, Sloane MS 963

London, British Library, Sloane MS 1314

London, British Library, Sloane MS 1754

London, British Library, Sloane MS 2457

London, British Library, Sloane MS 2584

London, British Library, Sloane MS 3542

London, British Library, Sloane MS 3564

London, Wellcome Historical Medical Library, MS 405

London, Wellcome Historical Medical Library, MS 542

Manchester, John Rylands University Library, English MS 404

Oxford, Bodleian Library, MS e Museo 219

Oxford, Bodleian Library, Ashmole MS 1447

Oxford, Bodleian Library, Rawlinson MS C.814

Paris, Bibliothèque Nationale de France, MS Nouvelles Acquisitions Latines 693

San Marino, Huntington Library, MS HM 58

San Marino, Huntington Library, MS HM 64

San Marino, Huntington Library, MS HM 1336

Stockholm Royal Library MS X.90

